# Supplementary material for: Genomic Insights and Antimicrobial Potential of Newly Streptomyces cavourensis Isolated from a Ramsar Wetland Ecosystem
Source: Microorganisms. 2025 Mar 3;13(3):576. doi: 10.3390/microorganisms13030576 (PMC11945845; doi:10.3390/microorganisms13030576)
Supplement: Supplementary file 1 [file microorganisms-13-00576-s001.zip › Table S1.pdf]

Table S1. Phenotypic characteristics and extracellular enzyme production of *Streptomyces* sp. ACT158 compared to the reference strain *S. cavourensis* NRBC 13026T (Skarbek and Brady, 1978) (+ Growth or positive reaction of the test strain, − no growth or negative reaction, ± average growth).

**Characteristics**		**ACT158**	***S. cavourensis* NRBC 13026^T**
**Growth / Diffusible pigments**			
ISP1		+++ / Brown	+++ / Brown
ISP2		+++ / Brown	+++ / Light brown
ISP3		-	+++ /None
ISP4		++ / None	++ /None
ISP5		+ / Light brown	+ /None
ISP6		+++ / Brown	++ Brown
ISP7		++ / Light brown	+++ Light brown
**Growth at :**			
NaCl (w/v)	0 %	+	+
	2.5 %	+	+/-
	5.0 %	+	-
Temperature	4°C	-	ND
	25°C	+	+
	30°C	+	+
	37°C	+	+
	42°C	+	ND
pH	4	+	ND
	5	+	+
	7	+	+
	9	+	ND
	10	+	ND
**Degradation of :**			
Gelatin		+	+
Esterase		+	-
Casein		+	+
Starch		+	+
Cellulose		+	+
**Utilization of :**			
Glucose		+	+
L-arabinose		+	+
Melibiose		+	ND
Rhamnose		+	-
Saccharose		+	ND
myso-inositol		+	-
Mannitol		+	+
Sorbitol		+	+
**Production of**			
Coagulase		+	ND
Nitrate reductase		+	ND
Urease		-	ND
Citrate permease		+	ND
Catalase		+	ND
Hemolytic activity		Type A	ND
