# Supplementary material for: Genomic Insights and Antimicrobial Potential of Newly Streptomyces cavourensis Isolated from a Ramsar Wetland Ecosystem
Source: Microorganisms. 2025 Mar 3;13(3):576. doi: 10.3390/microorganisms13030576 (PMC11945845; doi:10.3390/microorganisms13030576)
Supplement: Supplementary file 1 [file microorganisms-13-00576-s001.zip › Table S2.pdf]

Table S2. List of indicator microorganisms used in this study

Code	Organism	Species	Media
**StA1**	**Bacteria**	*Staphylococcus aureus* ATCC25293	Lureat Bertani
**StA2**		*Staphylococcus aureus* ATCC43300	Lureat Bertani
**MiL**		*Micrococcus luteus* DSM1790	Lureat Bertani
**EsC**		*Escherichia coli* ATCC25422	Lureat Bertani
**SaT**		*Salmonella* Typhimurium	Lureat Bertani
**KlP**		*Klebsiella pneumoniae*	Lureat Bertani
**BaS**		*Bacillus subtilis* ATCC6633	Lureat Bertani
**StA3**		*Staphylococcus aureus* SARM	Lureat Bertani
**EsC**		*Escherichia coli* DH5α	Lureat Bertani
**BT10**		*Pseudomonas aeruginosa*	Lureat Bertani
**Rlm**	**Yeast**	*Rhodotorula mucilaginosa*	Sabouraud
**Cpl**		*Candida parapscicola* ATCC22019	Sabouraud
**Cks**		Candida *krusei* ATCC6258	Sabouraud
**Cgl**		*Candida glabrata*	Sabouraud
**Cal**		*Candida albicans*	Sabouraud
**Sac**		*Saccharomyces* sp.	Sabouraud
**Kly**		*Kluveromyces* sp.	Sabouraud
**FusS**	**Filamentous**	*Fusarium solani*	Sabouraud
**LicC**	**fungi**	*Lichtheimia corymbifera*	Sabouraud
**LomP**		*Lomentospora prolificans*	Sabouraud
**AspC**		*Aspergillus calidostus*	Sabouraud
**FusO**		*Fusarium oxysporum*	Sabouraud
**AspF**		*Aspergillus fumugatus*	Sabouraud
**RhiO**		*Rhizopus oryzae*	Sabouraud
**PaeL**		*Paecilomyces lilium*	Sabouraud
**ApfL**		*Aspergillus flavus*	Sabouraud
**SceA**		*Scedosporium apiospermum*	Sabouraud
**ScoB**		*Scopilariopsis brevicaulis*	Sabouraud
**PenC**		*Penicillium chrysogenum*	Sabouraud
**ScoC**		*Scopulariopsis candida*	Sabouraud
**ArtV**		*Arthroderma vanbreuseghemii*	Sabouraud
**PecV**		*Pecilomyces variotii*	Sabouraud
