# Supplementary figures and images for: Genomic Insights and Antimicrobial Potential of Newly Streptomyces cavourensis Isolated from a Ramsar Wetland Ecosystem
Source: Microorganisms. 2025 Mar 3;13(3):576. doi: 10.3390/microorganisms13030576 (PMC11945845; doi:10.3390/microorganisms13030576)

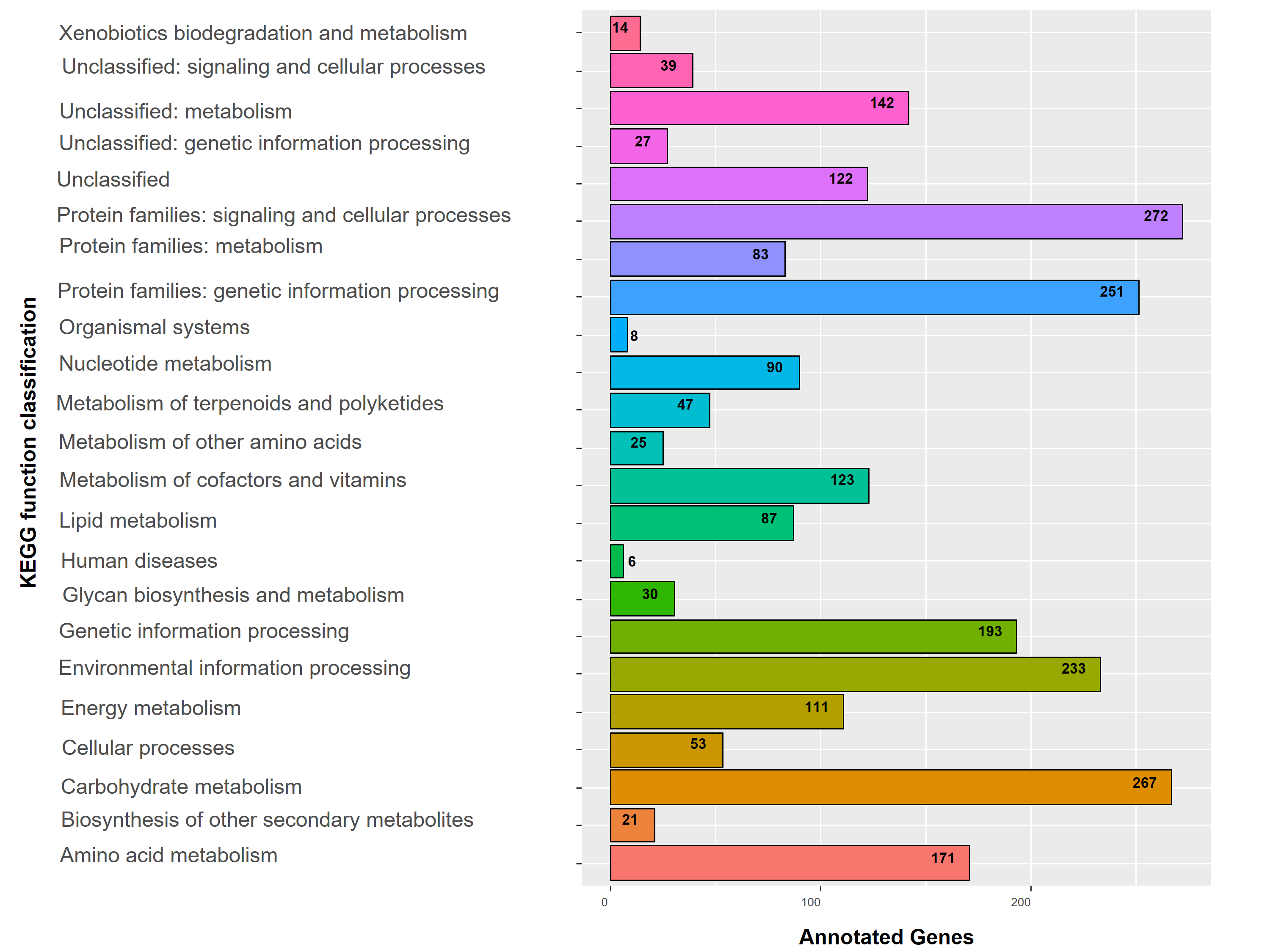

Supplement: Supplementary file 1 [file microorganisms-13-00576-s001.zip › Fig S2.TIF]

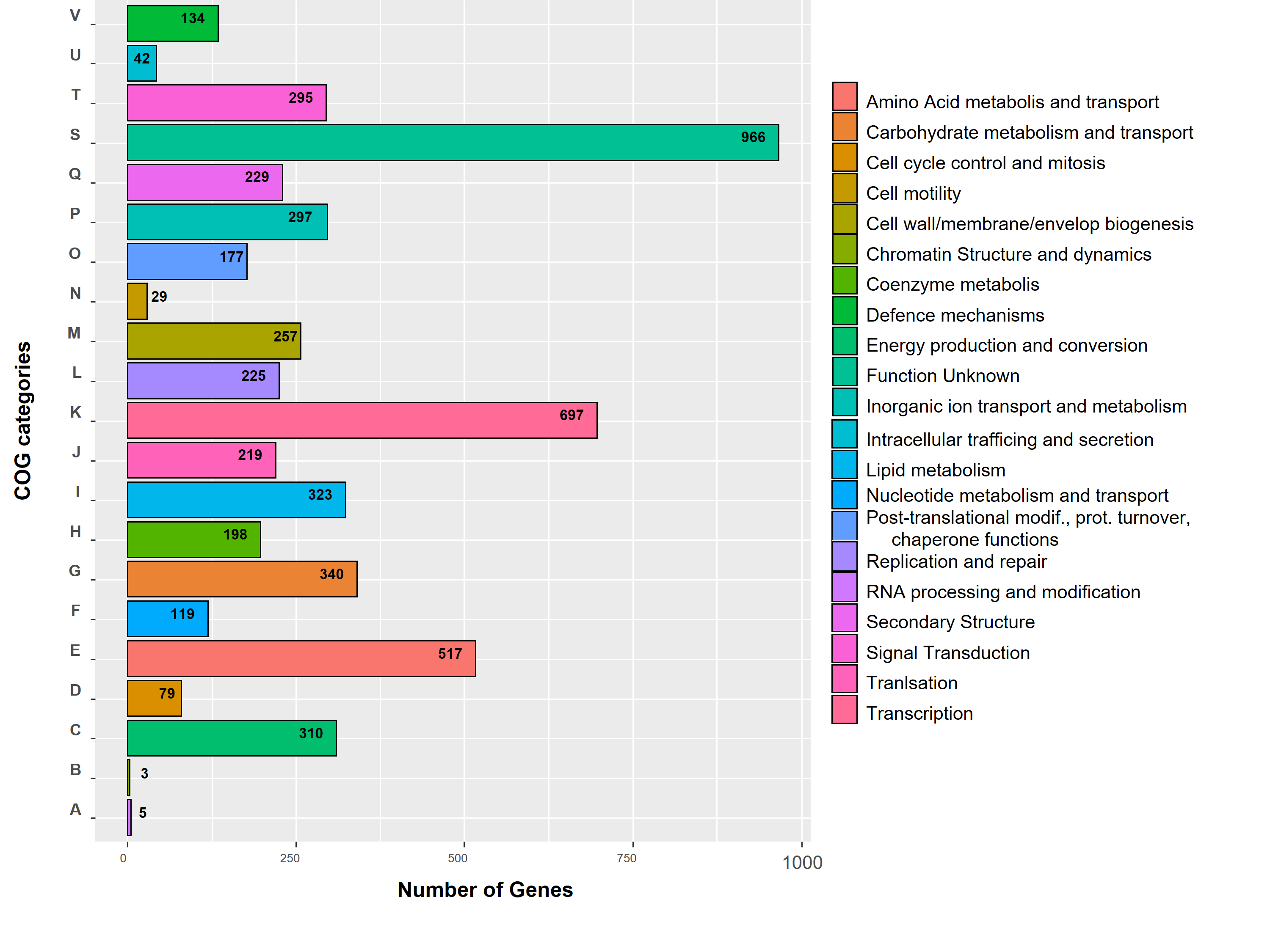

Supplement: Supplementary file 1 [file microorganisms-13-00576-s001.zip › Fig S3.TIF]

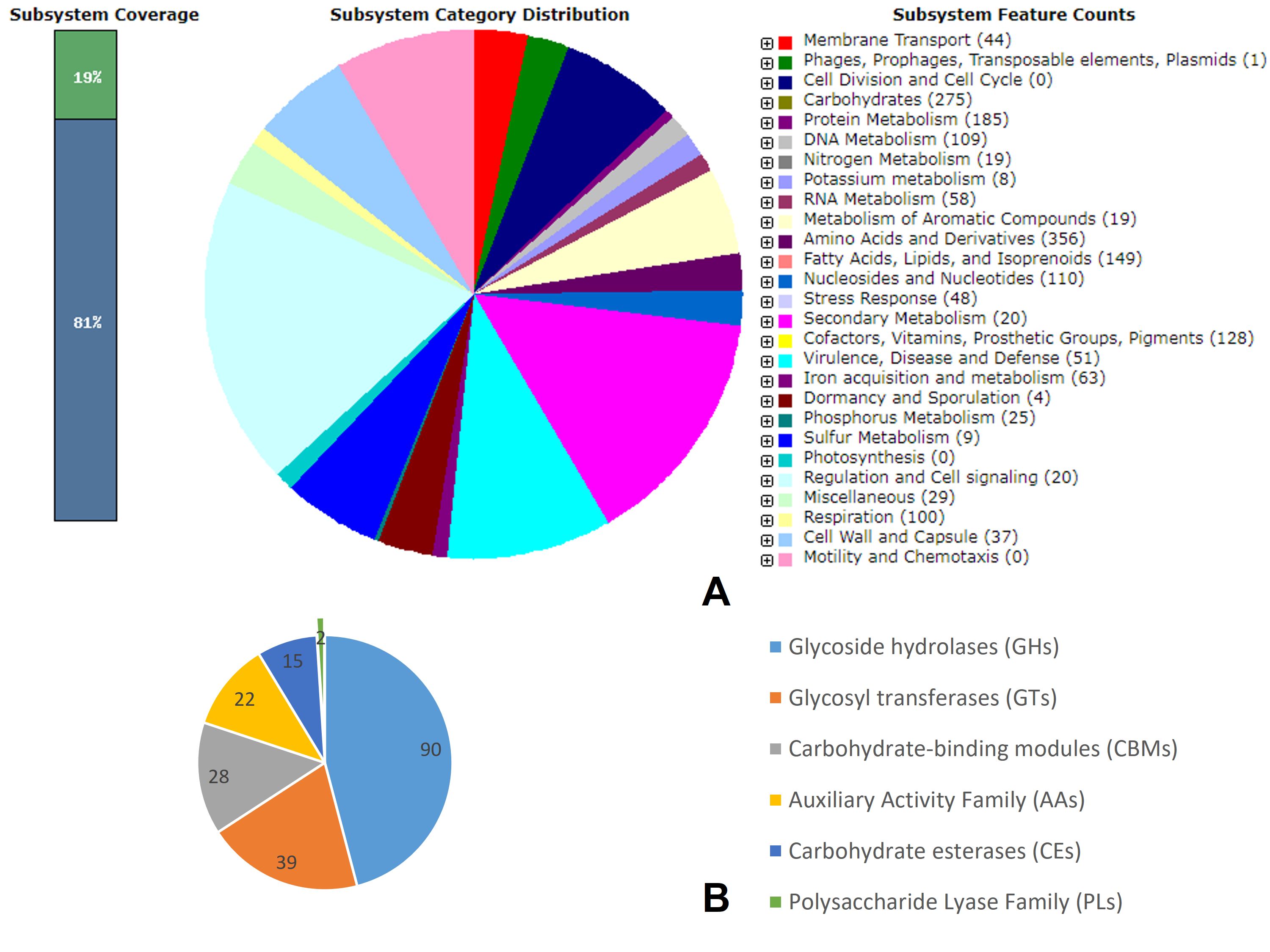

Supplement: Supplementary file 1 [file microorganisms-13-00576-s001.zip › Fig S4.TIF]

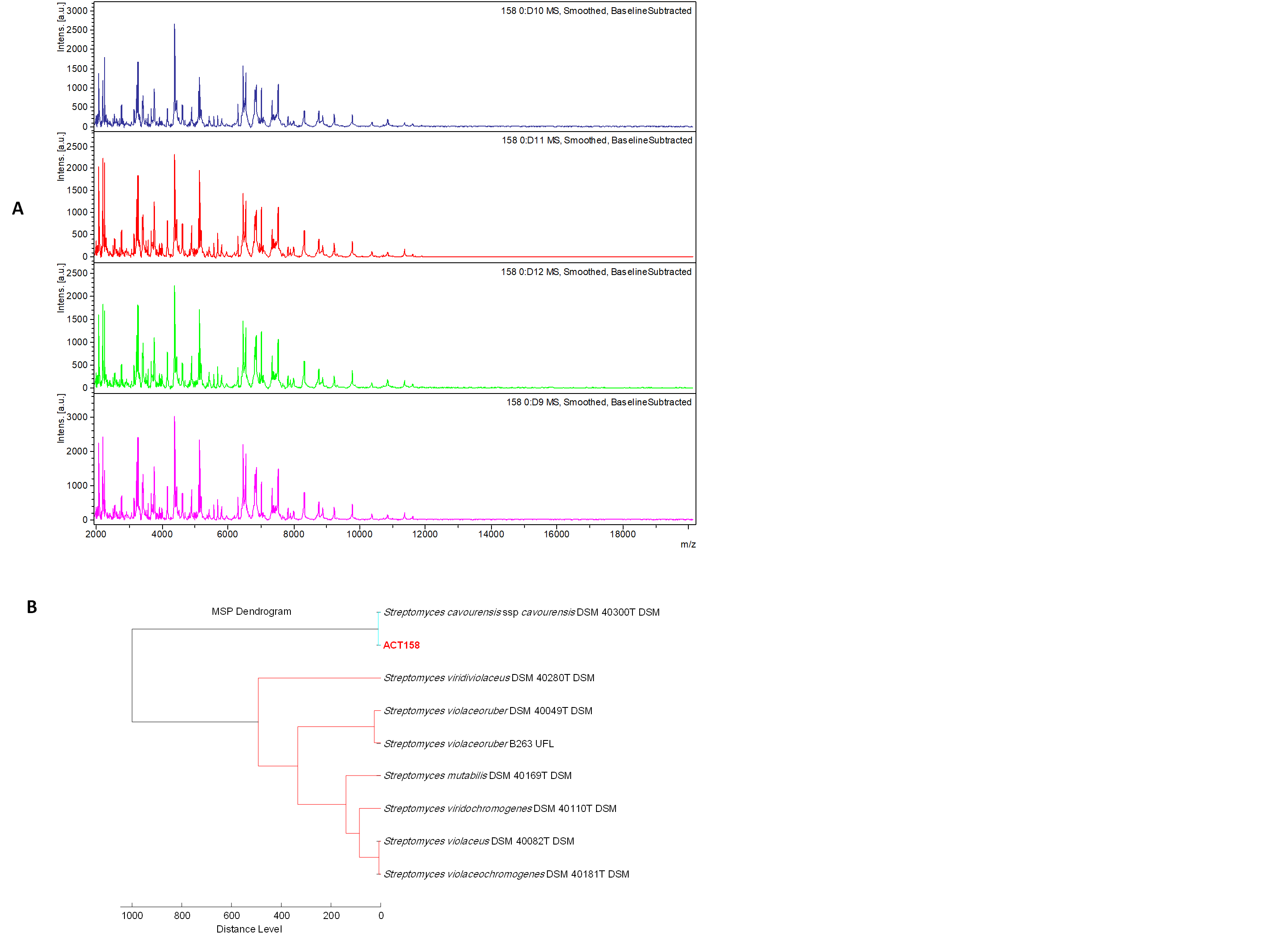

Supplement: Supplementary file 1 [file microorganisms-13-00576-s001.zip › Fig S1.tif]
